# R‐ketorolac ameliorates cancer‐associated cachexia and prolongs survival of tumour‐bearing mice

**DOI:** 10.1002/jcsm.13422

**Published:** 2024-02-01

**Authors:** Sophia E. Chrysostomou, Sandra Eder, Isabella Pototschnig, Anna‐Lena Mayer, Martina Derler, Marion Mussbacher, Silvia Schauer, Dongxu Zhang, Dongmei Yan, Gennie Liu, Gerald Hoefler, Thomas Weichhart, Paul W. Vesely, Lingbing Zhang, Martina Schweiger

**Affiliations:** ^1^ Institute of Molecular Biosciences University of Graz Graz Austria; ^2^ Institute of Pharmaceutical Sciences University of Graz Graz Austria; ^3^ Diagnostic and Research Institute of Pathology Medical University of Graz Graz Austria; ^4^ Yinuoke Ltd. Changchun China; ^5^ Department of Immunology Jilin University Changchun China; ^6^ BioTechMed‐Graz Graz Austria; ^7^ Institute of Medical Genetics Medical University of Vienna Vienna Austria; ^8^ Field of Excellence BioHealth ‐ University of Graz Graz Austria

**Keywords:** cachexia, cancer, inflammation, ketorolac

## Abstract

**Background:**

Cancer‐associated cachexia (CAC) is a debilitating syndrome associated with poor quality of life and reduced life expectancy of cancer patients. CAC is characterized by unintended body weight reduction due to muscle and adipose tissue loss. A major hallmark of CAC is systemic inflammation. Several non‐steroidal anti‐inflammatory drugs (NSAIDs) have been suggested for CAC treatment, yet no single medication has proven reliable. R‐ketorolac (RK) is the R‐enantiomer of a commonly used NSAID. The effect of RK on CAC has not yet been evaluated.

**Methods:**

Ten‐ to 11‐week‐old mice were inoculated with C26 or CHX207 cancer cells or vehicle control (phosphate‐buffered saline [PBS]). After cachexia onset, 2 mg/kg RK or PBS was administered daily by oral gavage. Body weight, food intake and tumour size were continuously measured. At study endpoints, blood was drawn, mice were sacrificed and tissues were excised. Immune cell abundance was analysed using a Cytek® Aurora spectral flow cytometer. Cyclooxygenase (COX) activity was determined in lung homogenates using a fluorometric kit. Muscle tissues were analysed for mRNA and protein expression by quantitative real‐time PCR and western blotting analysis, respectively. Muscle fibre size was determined on histological slides after haematoxylin/eosin staining.

**Results:**

Ten‐day survival rate of C26‐bearing animals was 10% while RK treatment resulted in a 100% survival rate (*P* = 0.0009). Chemotherapy resulted in a 10% survival rate 14 days after treatment initiation, but all mice survived upon co‐medication with RK and cyclophosphamide (*P* = 0.0001). Increased survival was associated with a protection from body weight loss in C26 (−0.61 ± 1.82 vs. −4.48 ± 2.0 g, *P* = 0.0004) and CHX207 (−0.49 ± 0.33 vs. −2.49 ± 0.93 g, *P* = 0.0003) tumour‐bearing mice treated with RK, compared with untreated mice. RK ameliorated musculus quadriceps (−1.7 ± 7.1% vs. −27.8 ± 8.3%, *P* = 0.0007) and gonadal white adipose tissue (−18.8 ± 49% vs. −69 ± 15.6%, *P* = 0.094) loss in tumour‐bearing mice, compared with untreated mice. Mechanistically, RK reduced circulating interleukin‐6 (IL‐6) concentrations from 334 ± 151 to 164 ± 123 pg/mL (*P* = 0.047) in C26 and from 93 ± 39 to 35 ± 6 pg/mL (*P* = 0.0053) in CHX207 tumour‐bearing mice. Moreover, RK protected mice from cancer‐induced T‐lymphopenia (+1.8 ± 42% vs. −49.2 ± 12.1% in treated vs. untreated mice, respectively). RK was ineffective in ameliorating CAC in thymus‐deficient nude mice, indicating that the beneficial effect of RK depends on T‐cells.

**Conclusions:**

RK improved T‐lymphopenia and decreased systemic IL‐6 concentrations, resulting in alleviation of cachexia and increased survival of cachexigenic tumour‐bearing mice, even under chemotherapy and independent of COX inhibition. Considering its potential, we propose that the use of RK should be investigated in patients suffering from CAC.

## Introduction

Cancer‐associated cachexia (CAC) is a devastating catabolic wasting syndrome that cannot be reversed by increased nutrient intake.^1^ The diagnostic criteria for cachexia in cancer patients, with a body mass index (BMI) ≥ 20 kg/m^2^, are unintended weight loss that is ≥5% within 6 months or ≥2% body weight loss and sarcopenia.^2^ Up to 80% of cancer patients suffer from CAC, and 25% of cancer deaths can be attributed to CAC. Unfortunately, chemotherapeutic agents, the primary treatment option for many types of cancer, aggravate the symptoms of cachexia.^3^ Consequently, patients in the refractory phase of CAC discontinue chemotherapy and are transferred to palliative care.

Systemic inflammation and increased abundance of circulating proinflammatory cytokines driving skeletal muscle atrophy, adipose tissue loss and anorexia are major hallmarks of CAC.^2^ Besides targeting specific inflammatory and/or catabolic cytokines, such as interleukin‐6 (IL‐6), common anti‐inflammatory drugs have been suggested for the treatment of CAC. With regard to weight loss and quality of life, beneficial effects have been shown for indomethacin, celecoxib and ibuprofen in cachectic patients.^4^ However, the prolonged use of these drugs is accompanied by noticeable side effects, raising concerns about their suitability in treating cachexia. Accordingly, despite its detrimental impact on life quality and prognosis, CAC is still an untreated disease.

Ketorolac (5‐benzoyl‐2,3‐dihydro‐1*H*‐pyrrolizine‐1‐carboxylic acid) is a non‐steroidal anti‐inflammatory drug (NSAID) that is widely used for opioid‐level pain management. Ketorolac is administered as a racemate, with the S‐form exhibiting inhibitory activity towards cyclooxygenase (COX) enzymes.^5^, ^6^ In cancer patients, ketorolac is used as an analgesic drug after surgeries and to control cancer‐associated pain. Its use is restricted to 5 days, due to renal and haematological toxicity of the S‐enantiomer.^7^ Compared with the S‐enantiomer, the R‐enantiomer of ketorolac shows 33‐fold lower ulcerogenic potential, presumably due to its inability to inhibit COX‐1 activity.^6^ Previous studies indicated that ketorolac increases ovarian‐,^8^ non–small‐cell lung‐,^9^ kidney‐ and breast cancer‐specific^10^ survival, which may be attributed to the ability of the R‐enantiomer to reduce tumour growth, invasion and metastasis via Rac1/Cdc42 inhibition.^11^ There is no study investigating the effect of ketorolac on CAC. Our work aimed at examining whether the R‐enantiomer of ketorolac (R‐ketorolac [RK]) prevents CAC in mice.

## Methods

### Cell culture

Cultured cells were kept at 37°C, 5% CO_2_, under a 95% humidified atmosphere. CHX207 fibrosarcoma,^12^ C26 (Amgen, Thousand Oaks, CA, USA) and 4T1 (AMSBIO, Cambridge, MA, USA) cancer cells were cultivated in Roswell Park Memorial Institute (RPMI) 1640 medium (#A10491‐01, Thermo Fisher Scientific, USA). All media were supplemented with 10% foetal bovine serum (FBS; Thermo Fisher Scientific), 100 IU/L of penicillin, 0.1 mg/L of streptomycin, 1 mM of sodium pyruvate (Invitrogen) and 1% non‐essential amino acids (Invitrogen).

### Animal studies

Mice were maintained under specific pathogen‐free conditions at housing temperatures of 21–23°C in a 14‐h light/10‐h dark cycle and fed a standard chow diet with ad libitum access to food and water. Ten‐ to 11‐week‐old C57BL/6J (Janvier Laboratories), Balb/c or nude mice (SPF Biotechnology Co., Ltd., Beijing, China) were injected with 1 × 10^6^ cancer cells or phosphate‐buffered saline (PBS) as vehicle control intramuscularly in the right hind leg. RK, anamorelin, cyclophosphamide (Cy) and NSC23766 were reconstituted in 1× PBS. A total of 2 mg/kg RK or 12 mg/kg anamorelin was administered in a total volume of 100 μL daily by oral gavage. A total of 120 mg/kg Cy and 4 mg/kg NSC23766 were administered by intraperitoneal (i.p.) injection. Onset of cachexia was defined by a body weight loss of ≥5% within 2–3 consecutive days (mild cachexia).^S1^ ^,^ ^12^ For animal welfare, experiments were discontinued before mice experienced clinically evident functional disorders. If mice underwent rapid weight loss of ≥20% within a short period, the experiment was terminated and mice were euthanized. At study endpoints, blood was drawn from the retro‐orbital plexus and mice were sacrificed by cervical dislocation. IL‐6 plasma concentrations were determined by enzyme‐linked immunosorbent assay (ELISA) (#88‐7064‐22, Invitrogen). Animal study protocols were approved by the Austrian Federal Ministry for Science, Research, and Economy (protocol numbers BMBWF‐66.007/0005‐V/3b/2019) and were conducted in compliance with the Council of Europe Convention (ETS 123) and guidelines established by the Animal Research Committee of Jilin University.

### Flow cytometry of spleen and blood

Blood analysis was performed by staining 5 μL of whole blood (collected in 12% acid citrate dextrose) with antibodies listed in the Supplementary Methods for 15 min. After fixation in 2% formaldehyde for 10 min, erythrocytes were lysed in lysis buffer (150 mM of NH_4_Cl, 10 mM of KHCO_3_ and 0.1 mM of Na_2_EDTA) for 10 min. Single‐cell suspensions from spleens were obtained by passing tissues through 70 μm cell strainers followed by erythrocyte lysis in lysis buffer. A total of 1 × 10^6^ cells were stained with antibodies for 20 min and fixed in 2% formaldehyde. Samples were immediately measured with a Cytek® Aurora spectral flow cytometer, and data were analysed with the SpectroFlow® and FlowJo® software.

### Cyclooxygenase‐activity assay

COX activity was determined in lung homogenates using a Fluorometric Cyclooxygenase Activity Assay (Abcam, ab204699) according to the manufacturer's protocol and as described in the Supplementary Methods.

### Muscle fibre analysis

Musculus quadriceps (m. quad) was excised, washed in 1× PBS, fixed in 4% formaldehyde and embedded in paraffin. Paraffin sections were stained with haematoxylin and eosin. The resulting slides were scanned and muscle fibre areas (>140 fibres per muscle) were analysed blinded using a CaseViewer application (3DHISTECH Ltd., Budapest, Hungary) as described before.^S2^

### Protein expression analysis

Muscle and adipose tissues were powdered on dry ice and disrupted in ice‐cold solution A (0.25 M of sucrose, 1 mM of EDTA and 1 mM of dithiothreitol, pH 7.0) supplemented with protease and phosphatase inhibitor (PhosSTOP, Sigma‐Aldrich, St. Louis, MO, USA) using an Ultra‐Turrax© tissue homogenizer. Homogenates were centrifuged for 30 min at 4°C and 16 000 × g, and protein concentration of the infranatant was determined by Protein Assay Dye (Bio‐Rad Laboratories, Hercules, CA, USA). Ten‐ to 15‐μg protein of the fat‐free tissue lysate were subjected to 10–12.5% sodium dodecyl sulfate‐polyacrylamide gel electrophoresis (SDS‐PAGE), and western blotting analysis was performed as described in the Supplementary Methods.

### Quantitative real‐time PCR analysis

Total tissue RNA was extracted using TRIzol (Invitrogen, Waltham, MA, USA), and cDNA was prepared using Luna Script RT Supermix Kit (NEB) according to the manufacturer's instructions. Quantitative real‐time (qRT) PCR was performed using StepOnePlus™ RT‐PCR System (Thermo Fisher Scientific) with SYBR Green (Bio‐Rad Laboratories). Primer sequences are listed in the Supplementary Methods.

### Statistical analysis

Data are shown as means with standard deviations (SDs). Statistical significances were determined by two‐tailed unpaired *t*‐test, one‐way analysis of variance (ANOVA) or two‐way ANOVA followed by Šidák's post hoc analysis using GraphPad Prism 8.0.1. Groups were considered significantly different at *P* ≤ 0.05. To determine the sample size required to test the study hypothesis, an a priori power analysis was conducted using ClinCalc.com. Results indicated that the required sample size to achieve 80% power for detecting a 10% effect with an effect size of 0.5 (control group weight: 25 ± 2 g), at a type I error rate of *α* = 0.05, was *n* = 7.

## Results

### R‐ketorolac treatment prolongs survival and alleviates C26‐induced weight loss independent of tumour growth and food intake

**Figure 1:**
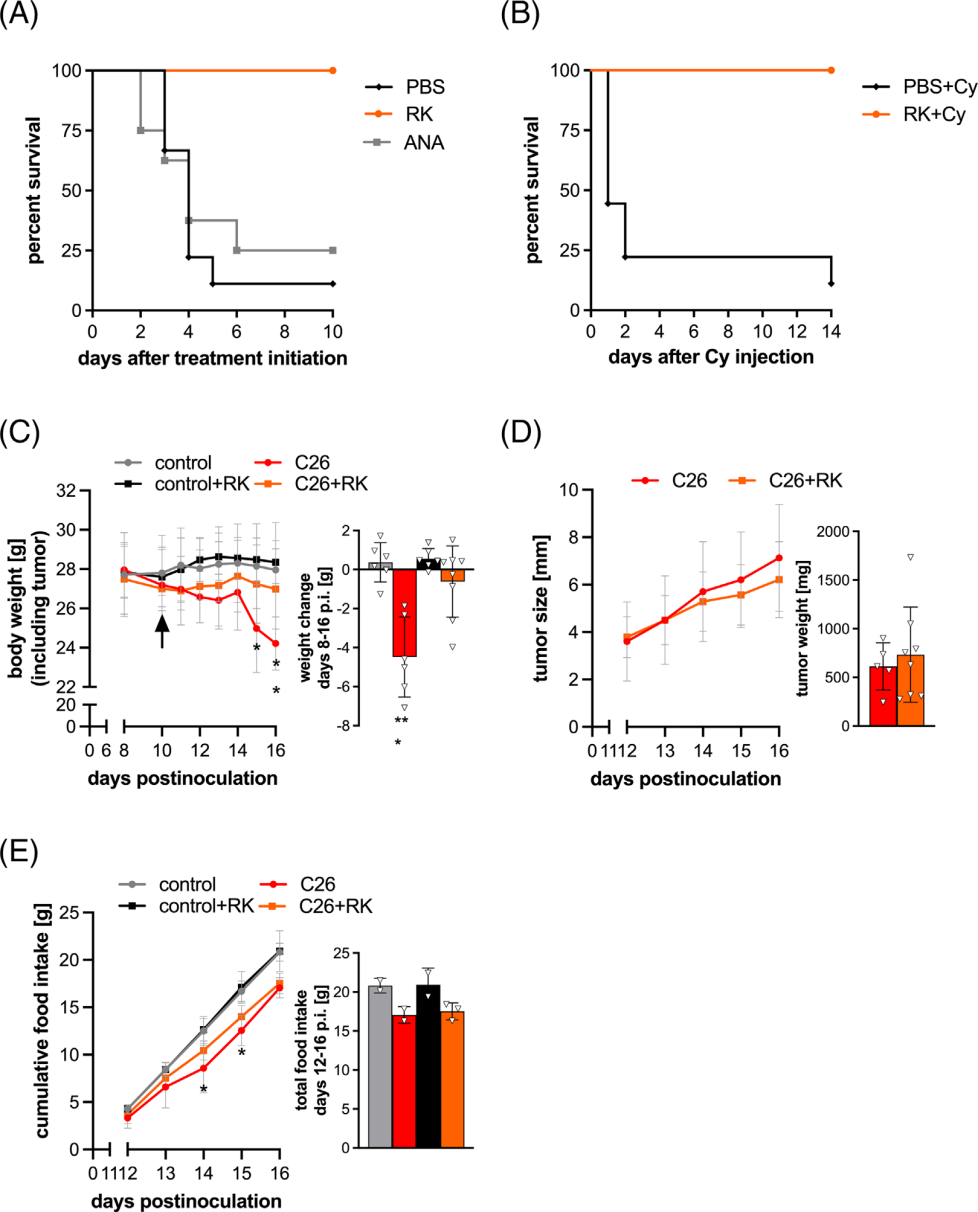
R‐ketorolac (RK) treatment prolongs survival and ameliorates cachexia without altering tumour growth or food intake in C26 tumour‐bearing mice. Balb/c mice were inoculated with 1 × 10^6^ C26 cells (in 100 μL of phosphate‐buffered saline [PBS]) or with PBS as control. (A) RK treatment prolongs survival of C26 tumour‐bearing mice. Upon cachexia onset (>5% body weight loss), mice were treated with 2 mg/kg RK (*n* = 9) or 12 mg/kg anamorelin (ANA; *n* = 8) daily via oral gavage for 10 consecutive days. Mice treated with PBS were used as control (*n* = 9). Survival after treatment initiation was recorded and plotted into Kaplan–Meier survival curves (*P* = 0.0009, log‐rank test, according to Mantel–Cox). (B) RK enhances chemotherapy tolerance of C26 tumour‐bearing mice. Upon cachexia onset (>5% body weight loss), mice were treated with 2 mg/kg RK (*n* = 9) or PBS (*n* = 9) daily. After body weight recovery of mice in the RK‐treated group, mice were injected intraperitoneally (i.p.) with 120 mg/kg cyclophosphamide (Cy). After recovery from Cy‐induced body weight loss, a second Cy injection was given to both groups. A total of three injections of Cy were given if mice survived previous injections. Survival time after first Cy injection was recorded and plotted into Kaplan–Meier survival curves (*P* = 0.0001, log‐rank test, according to Mantel–Cox). (C–E) Starting from Day 8 after cancer cell inoculation (Day 8 p.i.), body weight, tumour size and food intake of mice were monitored regularly. On Day 10 p.i. (indicated with an arrow), mice were daily treated with 2 mg/kg RK or PBS as control (*n* = 6–8). (C) Body weight change over time (including tumour weight) and total weight change within 8 days (tumour weight subtracted). (D) Tumour growth over time was determined using a sliding calliper. At study endpoints, tumours were excised and weighed. (E) Food intake was determined by weighing the amount of food in the food hopper every day and is shown as cumulative food intake over time and as total food intake between days 12 and 16 p.i. (C–E) Two‐way analysis of variance (ANOVA) (curves) or one‐way ANOVA (bar graphs) followed by Šidák's post hoc analyses was performed to identify statistical differences between the groups (*P ≤ 0.05; **P ≤ 0.01; ***P ≤ 0.001, *control vs. tumour‐bearing mice).

To study the effects of RK treatment in cachectic tumour‐bearing mice, we orally administered 2 mg/kg RK to C26 tumour‐bearing mice (C26 mice) every day after cachexia onset (≥5% body weight loss). As controls, we daily administered anamorelin or PBS (vehicle). Ten per cent of PBS‐treated and 25% of anamorelin‐treated animals survived 10 days after treatment initiation. In contrast, all RK‐treated mice were still alive 10 days after treatment initiation (*P* = 0.0009) (*Figure* *1* *A*). Next, to explore whether RK treatment is compatible with chemotherapy, we treated cachectic mice with RK and the chemotherapeutic drug cyclophosphamide (Cy). While 90% of the animals receiving only Cy reached a humane endpoint within 14 days after treatment start, survival rate for Cy‐ and RK‐treated animals was 100% (*P* = 0.0001), evidently pointing towards the therapeutic potential of the drug for patients receiving anti‐cancer treatment (*Figure* *1* *B*).

To study how RK treatment mediates prolonged survival of C26 mice, we monitored body weight, tumour growth and food intake of C26 and non‐tumour‐bearing (control) mice that were treated with RK or PBS as vehicle control after cachexia onset. PBS‐treated C26 mice were sacrificed on Days 16 and 17 post cancer cell inoculation (p.i.) with a tumour weight of 0.6 g and a weight loss of 4.48 ± 2.0 g (−16%) (*Figure* *1* *C,E*). C26 mice treated with RK were sacrificed with a tumour weight of 0.7 g and a weight loss of 0.61 ± 1.82 g (−2.2%) (*Figure* *1* *C,D*). RK had no effect on body weight of control mice or non‐cachexigenic 4T1‐bearing mice (*Figures* *1* *C* and *S1* *A,B*). Tumour growth was comparable between PBS‐treated and RK‐treated C26 mice (*Figure* *1* *D*). To decipher whether RK protects from weight loss by increasing caloric uptake, we measured food intake between days 12 and 16 p.i. Total food intake was similarly reduced in PBS‐ and RK‐treated C26 mice (17.1 ± 0.8 vs. 17.5 ± 1.1 g, respectively) compared with controls and not different between PBS‐ and RK‐treated control mice (20.8 ± 0.7 vs. 20.9 ± 1.5 g, respectively), indicating no effect of RK on food intake (*Figure* *1* *E*). Hence, RK treatment protects from C26‐induced weight loss, independent of tumour growth and food intake.

Previous studies reported that RK is inactive towards COX‐1 or COX‐2 but inhibits Rac1.^6^, ^11^ To verify that RK does not inhibit COX enzymes, we orally administered 2 mg/kg RK to mice for 5 consecutive days and determined COX activity in lung homogenates. COX inhibition (COXi) by SC560 and celecoxib similarly reduced fluorescence intensity units (RFU) in lungs of PBS‐ and RK‐treated mice (*Figure* *S2B*). We found no difference in total COX activity between PBS‐ and RK‐treated mice (*Figure* *S2A*). To investigate whether Rac1 inhibition exerted similar beneficial effects on CAC as RK, we treated C26 mice daily with NSC23766 or RK starting 2 days after cachexia onset (*Figure* *S2C,D*). Using this treatment regimen, NSC23766 treatment slightly delayed but did not halt severe body weight loss in C26 mice (*Figure* *S2C*). In contrast, RK‐treated C26 mice already gained weight after 1 day and stayed weight stable thereafter (*Figure* *S2D*). Together, these results indicate that RK increases survival and prevents weight loss of C26 mice independent of COX and Rac1.

### R‐ketorolac treatment ameliorates adipose and muscle loss and impairs tissue signal transducer and activator of transcription 3 signalling in C26 mice

**Figure 2:**
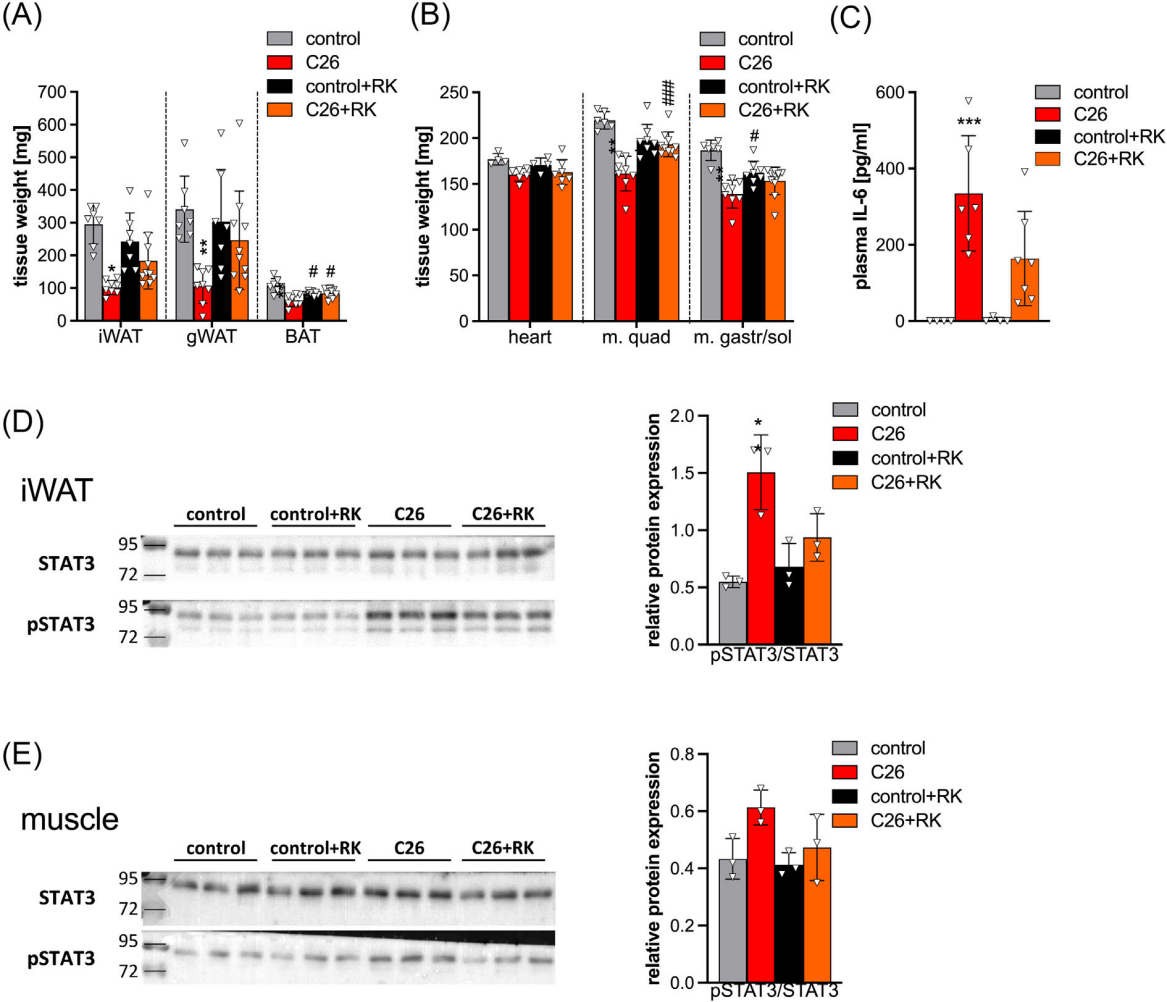
R‐ketorolac (RK) treatment ameliorates C26‐induced adipose and muscle loss by impairing tissue signal transducer and activator of transcription 3 (STAT3) signalling. Twelve days after C26 cancer cell inoculation and 2 days of treatment with 2 mg/kg RK or phosphate‐buffered saline (PBS) as control, blood was taken retro‐orbitally, mice were sacrificed and tissues were weighed and frozen (*n* = 5–8 for all control and *n* = 6–10 for all C26‐bearing mice). (A) Adipose tissue weight (inguinal white adipose tissue [iWAT], gonadal white adipose tissue [gWAT] and brown adipose tissue [BAT]) and (B) muscle tissue weight (heart, musculus quadriceps [m. quad] and m. gastrocnemius/m. soleus [m. gastr/sol]) of mice. (C) Plasma interleukin‐6 (IL‐6) concentration was determined using enzyme‐linked immunosorbent assay (eBioscience). (D, E) Western blotting analysis of muscle and adipose tissues. Signal intensities were determined using ChemiDoc (Bio‐Rad) and calculated using Image Lab (Bio‐Rad). One‐way analysis of variance followed by Šidák's post hoc analysis was performed to identify statistical differences between the groups (*^/#^ *P* ≤ 0.05; **^/##^ *P* ≤ 0.01; ***^/###^ *P* ≤ 0.001, *control vs. C26 and control + RK vs. C26 + RK; ^#^control vs. control + RK and C26 vs. C26 + RK).

To investigate the mechanisms by which RK prevents weight loss, we analysed tissues of control and C26 mice 12 days p.i. (≤10% body weight loss) that were treated with RK or PBS for 2 days. Twelve days p.i., PBS‐treated C26 mice exhibited reduced adipose tissue weight (inguinal white adipose tissue [iWAT]: −65.4 ± 7.3%; gonadal white adipose tissue [gWAT]: −69 ± 15.6%; and interscapular brown adipose tissue [BAT]: −43 ± 16.6%) compared with control mice (*Figure* *2* *A*). RK treatment ameliorated the reduction of adipose tissue in C26 mice (iWAT: −24 ± 35%; gWAT: −18.8 ± 49%; and BAT: no reduction compared with RK‐treated controls) (*Figure* *2* *A*). Tissue weight of cardiac muscle, m. quad and musculus gastrocnemius/soleus (m. gastr/sol) was reduced by 9.6 ± 3.9%, 27.8 ± 8.3% and 25 ± 8.7%, respectively, in C26 mice compared with control mice (*Figure* *2* *B*). Lower muscle weights were associated with 13.5% reduced m. quad fibre cross‐sectional area (*Figure* *S3A*) and a shift towards smaller muscle fibres (50% < 1500 cm^2^) in C26 mice compared with control mice (36% < 1500 cm^2^) (*Figure* *S3B*). Although RK‐treated mice exhibited reduced muscle weight (m. quad and m. gastr/sol: −10 ± 8% and −13.1 ± 6.8%, respectively) compared with PBS‐treated mice, RK ameliorated muscle loss in C26 mice (−4.5 ± 8%, −1.7 ± 7.1% and −4.3 ± 9.1% of heart, m. quad and m. gastr/sol, respectively) (*Figure* *2* *B*). However, reduced muscle loss was not associated with an increase in muscle fibre cross‐sectional area in RK‐treated C26 mice (*Figure* *S3A,B*). To delineate a potential mechanism for increased muscle weight in RK‐treated C26 mice, we performed expression analysis of atrophy marker genes. Western blotting analysis revealed that microtubule‐associated protein 1A/1B‐light chain 3 (LC3BII), a marker for autophagy, was increased in muscles of C26 mice (two‐fold) and reduced by 55% in RK‐treated C26 mice compared with untreated C26 mice (*Figure* *S3C*). Likewise, mRNA expression of *Fbxo32*, coding for Atrogin‐1, and *Trim63*, coding for MuRF1, the main ubiquitin ligases responsible for marking proteins for proteasomal degradation, was highly upregulated (3.6‐fold and 4‐fold, respectively) in skeletal muscle of C26‐bearing mice but not in RK‐treated C26 mice (*Figure* *S3D,E*). This indicates that RK ameliorates C26‐induced muscle wasting by preventing protein degradation.

In C26‐associated cachexia, it is well established that tissue atrophy is largely driven by IL‐6‐mediated activation of the transcription factor signal transducer and activator of transcription 3 (STAT3).^13^ We also detected high circulating IL‐6 concentrations in C26 mice (334 ± 151 pg/mL), which were reduced to 164 ± 123 pg/mL in RK‐treated C26 mice (*Figure* *2* *C*). IL‐6 was not detected in plasma of control mice. Elevated IL‐6 plasma concentrations translated into 2.7‐fold and 1.5‐fold increased pSTAT3 levels in adipose tissue and muscles of C26 mice compared with controls, respectively (*Figure* *2* *D,E*). RK treatment reversed C26‐induced pSTAT3 back to control levels in adipose tissue and muscle tissue (*Figure* *2* *D,E*). Together, these data indicate that RK prevents severe tissue atrophy in C26‐induced cachexia by reducing circulating IL‐6 concentrations and STAT3 signalling.

### R‐ketorolac does not affect C26‐associated hepatosplenomegaly and splenic lymphocyte numbers

**Figure 3:**
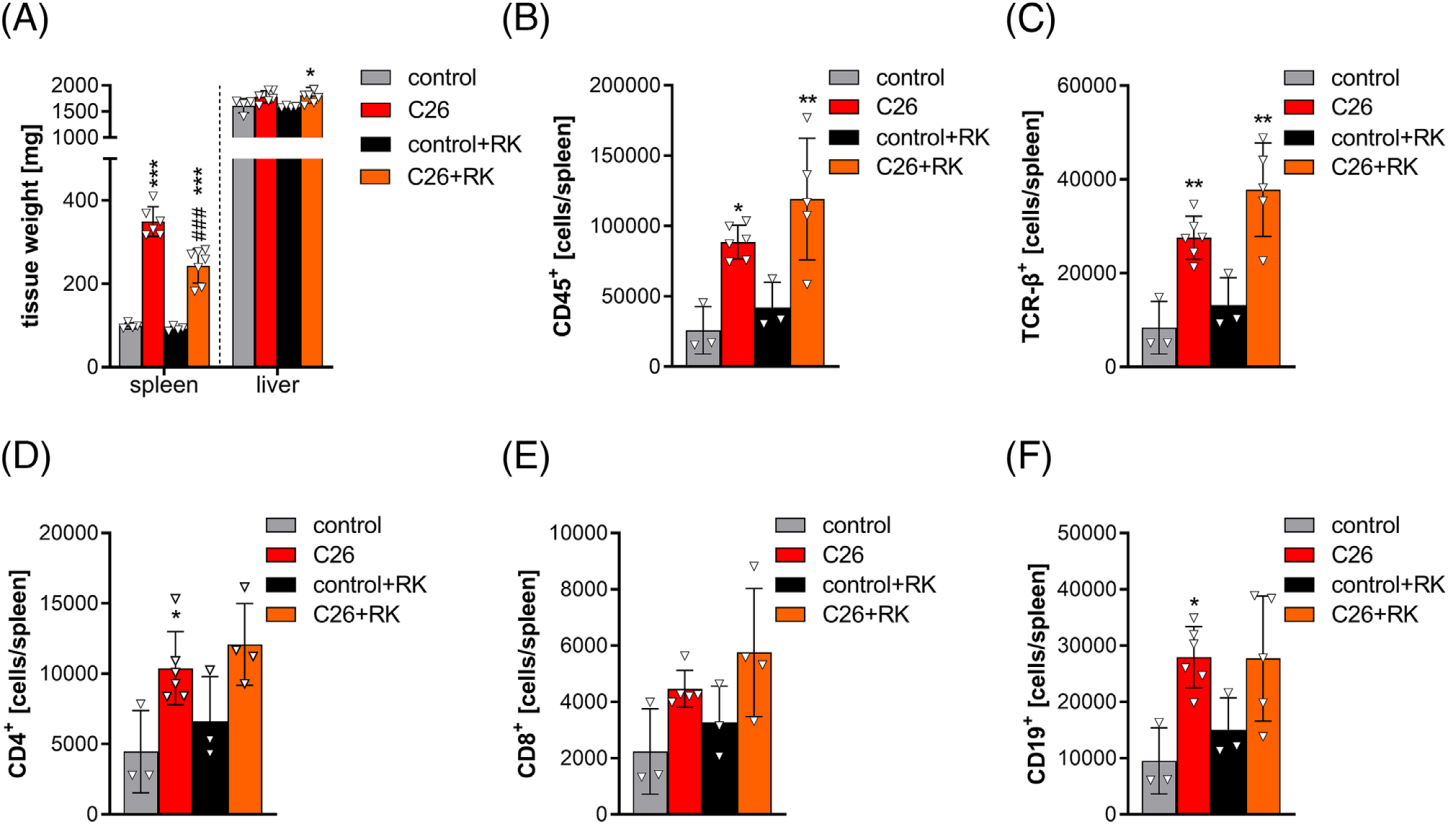
R‐ketorolac (RK) does not affect C26‐associated hepatosplenomegaly and splenic immune cell abundance. Twelve days after C26 cancer cell inoculation and treatment with 2 mg/kg RK or phosphate‐buffered saline (PBS) as control, blood was taken retro‐orbitally, mice were sacrificed and tissues were weighed and frozen. (A) Spleen and liver weight of mice (*n* = 6–7 for C26 and *n* = 4 for control). (B–F) Flow cytometry analysis of splenic immune cells in C26 mice. Single cells from spleens were stained using the indicated antibodies and analysed using a Cytek® Aurora spectral flow cytometer (*n* = 3 for control and *n* = 5–6 for C26 mice). One‐way analysis of variance followed by Šidák's post hoc analysis was performed to identify statistical differences between the groups (*^/#^ *P* ≤ 0.05; **^/##^ *P* ≤ 0.01; ***^/###^ *P* ≤ 0.001, *control vs. C26 and control + RK vs. C26 + RK; ^#^control vs. control + RK and C26 vs. C26 + RK).

Hepatosplenomegaly was evident by 3.6‐fold and 2.6‐fold increased spleen weight and 1.1‐fold increased liver weight in PBS‐ and RK‐treated C26 mice, respectively, compared with the respective control mice (*Figure* *3* *A*). Being a secondary lymphoid organ, the spleen is the site for maturation and storage of lymphocytes and splenomegaly is often associated with splenic hyperfunction and elevated immune cell turnover.^S3^ We performed flow cytometry analyses of splenic immune cells and found that CD45^+^ cells increased 3.4‐fold in PBS‐treated C26 mice and 2.8‐fold in RK‐treated C26 mice compared with the respective control mice (*Figure* *3* *B*). Similarly, cells positive for the T‐cell receptor‐β (TCR‐β^+^) were increased in spleens of PBS‐treated C26 mice (3.3‐fold) and C26 mice treated with RK (2.9‐fold) compared with the respective control mice (*Figure* *3* *C*). A trend towards increased numbers of CD4^+^ (T‐helper) and CD8^+^ (T‐killer) cells was observed in PBS‐ and RK‐treated C26 mice, compared with controls (*Figure* *3* *D,E*). Moreover, CD19^+^ cells, marking B‐lymphocytes, were increased three‐fold in spleens of PBS‐ and RK‐treated C26 mice compared with controls (*Figure* *3* *F*). These results demonstrate increased B‐ and T‐lymphocyte abundance in spleens of C26 mice, which is not altered upon RK treatment.

### The effect of R‐ketorolac is T‐cell dependent

**Figure 4:**
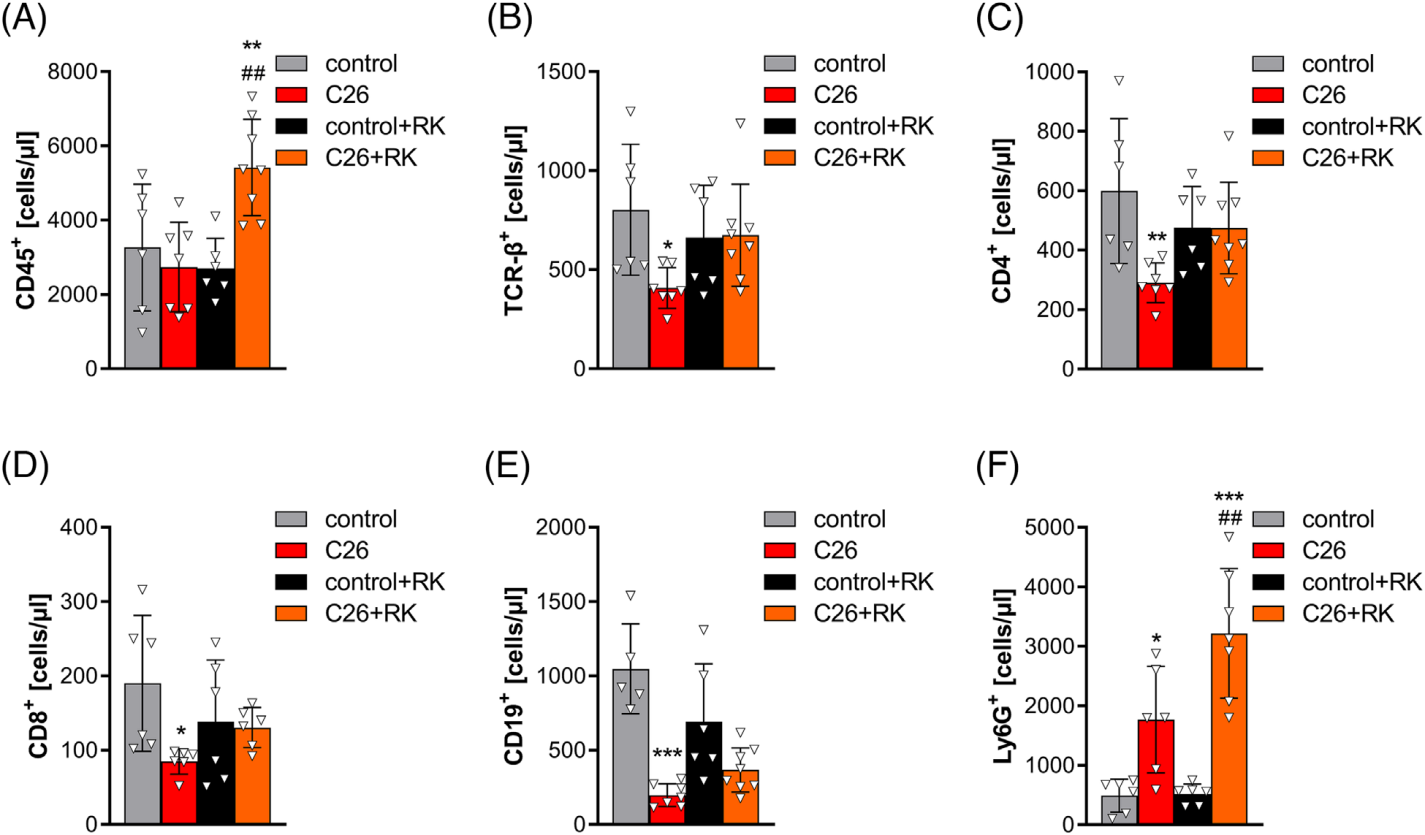
R‐ketorolac (RK) prevents C26‐induced T‐lymphopenia. Twelve days after C26 cancer cell inoculation and treatment with phosphate‐buffered saline (PBS) as control or 2 mg/kg RK, blood was taken retro‐orbitally and mice were sacrificed. (A–F) Whole blood (anticoagulated with acid citrate dextrose) was stained using the indicated antibodies and analysed using a Cytek® Aurora spectral flow cytometer (*n* = 6 for control and *n* = 7–8 for C26 mice). One‐way analysis of variance followed by Šidák's post hoc analysis was performed to identify statistical differences between the groups (*^/#^ *P* ≤ 0.05; **^/##^ *P* ≤ 0.01; ***^/###^ *P* ≤ 0.001, *control vs. C26 and control + RK vs. C26 + RK; ^#^control vs. control + RK and C26 vs. C26 + RK).

To get further insight into the immune cell status of cachectic animals, we performed flow cytometry analyses of whole blood (*Figure* *4* *A–F*). CD45^+^ cells were increased two‐fold in the blood of RK‐ but not PBS‐treated C26 mice compared with their respective controls (*Figure* *4* *A*). TCR‐β^+^, CD4^+^ and CD8^+^ T‐cells were decreased by 49.2 ± 12.1%, 51.5 ± 10.3% and 55.3 ± 8.2%, respectively, in the blood of PBS‐treated C26 mice compared with control mice. Although RK treatment per se slightly reduced TCR‐β^+^, CD4^+^ and CD8^+^ cell numbers, no reduction of T‐cells was observed in RK‐treated C26 mice compared with RK‐treated control mice (*Figure* *4* *B–D*). Similar to T‐cells, the number of B‐lymphocytes (CD19^+^ cells) was reduced in the blood of C26 mice compared with control mice (−81.1 ± 6.7%) (*Figure* *4* *E*). Although there was a slight increase compared with PBS‐treated C26 mice, RK‐treated C26 mice also had reduced CD19^+^ cells (−48 ± 20.9%) in the blood compared with RK‐treated control mice (*Figure* *4* *E*). Ly6G^+^ cells, marking peripheral neutrophils, were elevated 3.6‐fold in the circulation of PBS‐treated C26 mice, which was exacerbated upon RK treatment (6.3‐fold) compared with the respective controls (*Figure* *4* *F*). Together, and in accordance with previous data,^14^ our results point towards lymphopenia (B‐ and T‐cell reduction) and neutrophilia in C26 mice. T‐lymphopenia but not neutrophilia was ameliorated upon RK treatment.

**Figure 5:**
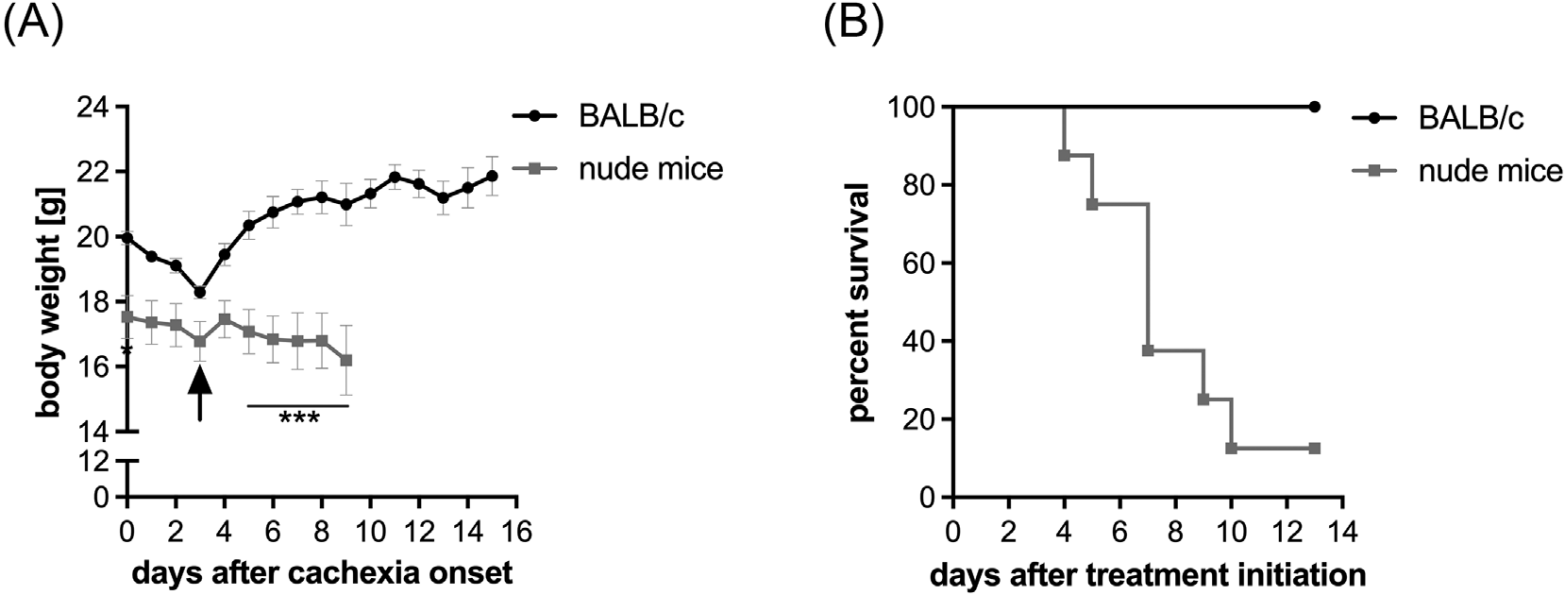
The effect of R‐ketorolac (RK) is T‐cell dependent. Balb/c or nude mice were inoculated with 1 × 10^6^ C26 cells. Three days after cachexia onset, mice were treated with 2 mg/kg RK or phosphate‐buffered saline (PBS) as control daily (indicated with an arrow) (*n* = 8). (A) Body weight curve of C26 tumour‐bearing Balb/c and nude mice. Two‐way analysis of variance followed by Šidák's post hoc analysis was performed to identify statistical differences between the groups (***P ≤ 0.001). (B) Kaplan–Meier plot to depict survival rate of tumour‐bearing mice (*P* = 0.0002, log‐rank test, according to Mantel–Cox).

To find out whether the RK effect on cachexia is T‐cell dependent, we investigated treatment efficacy in nude mice. Because they lack a thymus, nude mice cannot generate mature T‐lymphocytes. We started daily RK treatment of C26‐bearing Balb/c and nude mice 3 days after cachexia onset. RK reversed body weight loss only in Balb/c but not in nude mice. Accordingly, survival rate of C26‐bearing Balb/c mice was 100%, while 87.5% of C26‐bearing nude mice reached a humane endpoint 10 days after treatment initiation (*P* = 0.0002) (*Figure* *5* *A,B*). This indicates that RK exerts its protective effect by increasing circulating T‐lymphocytes in lymphopenic C26 mice.

### R‐ketorolac ameliorates cachexia in fibrosarcoma‐bearing mice

**Figure 6:**
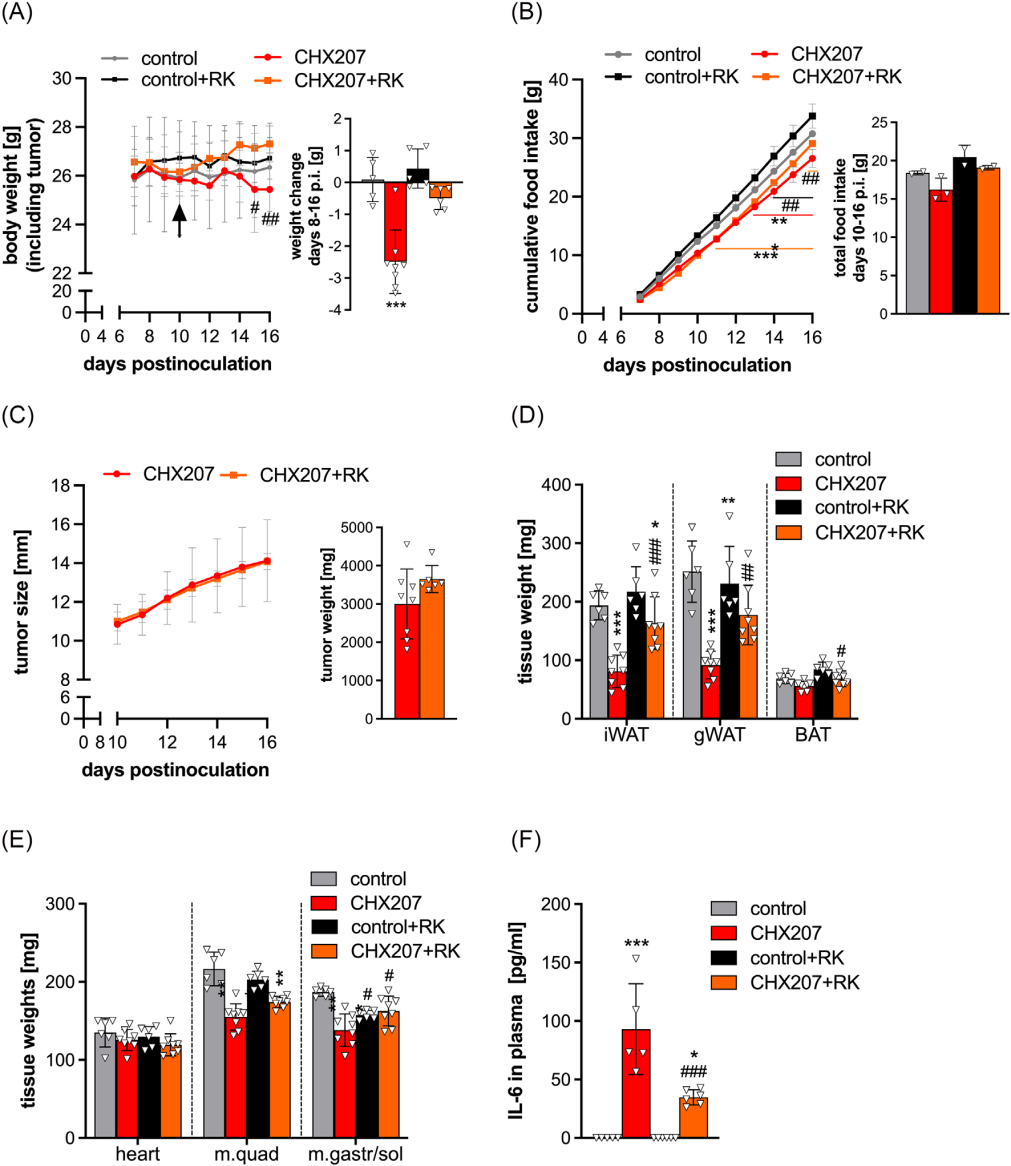
R‐ketorolac (RK) ameliorates cachexia in fibrosarcoma‐bearing mice. Male C57BL/6J mice were inoculated with 1 × 10^6^ CHX207 cells (in 100 μL of phosphate‐buffered saline [PBS]) or PBS as control. Starting from Day 7 after inoculation (Day 7 p.i.), food intake, body weight and tumour size were monitored regularly. Upon cachexia onset (>5% body weight loss), mice were treated with 2 mg/kg RK daily by oral gavage (*n* = 6–8 for CHX207 mice and *n* = 5 for control mice). (A) Body weight change over time (including tumour weight) and total weight change within 8 days (tumour weight subtracted). (B) Food intake was determined by weighing the amount of food in the food hopper every day and is shown as cumulative food intake over time and as total food intake between Days 10 and 16 p.i. (C) Tumour growth over time was determined using a sliding calliper. At study endpoints, tumours were excised and weighed. (D–F) Blood was taken retro‐orbitally, mice were sacrificed and tissues were weighed and frozen. (D) Adipose tissue weight (inguinal white adipose tissue [iWAT], gonadal white adipose tissue [gWAT] and brown adipose tissue [BAT]) and (E) muscle tissue weight (heart, musculus quadriceps [m. quad] and m. gastrocnemius/m. soleus [m. gastr/sol]) of mice. (F) Plasma interleukin‐6 (IL‐6) concentration was determined using enzyme‐linked immunosorbent assay (eBioscience). Two‐way analysis of variance (ANOVA) (curves) or one‐way ANOVA (bar graphs) followed by Šidák's post hoc analyses was performed to identify statistical differences between the groups (*^/#^ *P* ≤ 0.05; **^/##^ *P* ≤ 0.01; ***^/###^ *P* ≤ 0.001, *control vs. C26 and control + RK vs. C26 + RK; ^#^control vs. control + RK and C26 vs. C26 + RK).

To study the effect of RK treatment on cachexia development in a second cancer model and in mice with a different genetic background, we treated CHX207‐fibrosarcoma‐bearing C57BL/6J mice (CHX207 mice) with RK or PBS daily, starting at a body weight loss of approximately 5% (Day 10 p.i.). While PBS‐treated CHX207 mice continuously lost body weight, RK treatment caused an increase in body weight of CHX207 mice (*Figure* *6* *A*). This increase in body weight was mainly due to tumour growth, as tumours of CHX207 mice and RK‐treated CHX207 mice weighed 3.00 ± 0.91 and 3.59 ± 0.35 g, respectively (*Figure* *6* *C*). Upon subtraction of tumour weight, we found that CHX207 mice lost 2.49 ± 0.93 g of body weight while RK‐treated CHX207 mice lost only 0.49 ± 0.33 g within 8 days (Days 8–16 p.i.) (*Figure* *6* *A*). RK did not affect body weight of control mice. Cumulative food intake was reduced in RK‐ and PBS‐treated CHX207 mice compared with the respective controls (*Figure* *6* *B*). In total, RK treatment slightly increased food intake in control mice (18.4 ± 0.24 vs. 20.5 ± 1.6 g of untreated vs. treated mice, respectively) and CHX207 mice (16.2 ± 1.5 vs. 19.1 ± 0.28 g of untreated vs. treated mice, respectively) from Days 10 to 16 p.i. (*Figure* *6* *B*). Tumour growth was comparable between the groups (*Figure* *6* *C*). iWAT, gWAT and BAT weight was significantly reduced by 58.1 ± 14.2%, 63.4 ± 9.4% and 19 ± 11.4% (not significant), respectively, in CHX207 mice compared with control mice (*Figure* *6* *D*). RK treatment ameliorated cancer‐induced adipose tissue loss in CHX207 mice (iWAT: −20.6 ± 10.7%; gWAT: −23.1 ± 20.1%; and BAT: −15.1 ± 10.2%) and increased iWAT and gWAT weight by 2‐fold and 1.9‐fold, respectively, compared with PBS‐treated CHX207 mice (*Figure* *6* *D*). No differences in heart weight were observed between the groups (*Figure* *6* *E*). Tissue weight of m. quad and m. gastr/sol was significantly reduced by 28.4 ± 7.8% and 26.1 ± 11%, respectively, in CHX207 mice compared with control mice (*Figure* *6* *E*). RK treatment ameliorated cancer‐induced muscle loss in CHX207 mice (m. quad: −14.1 ± 3.5%; and m. gastr/sol: +3.2 ± 11%) (*Figure* *6* *E*). These results suggest that RK slightly increases food intake and ameliorates body weight loss and tissue wasting independent of tumour growth in CHX207 mice.

**Figure 7:**
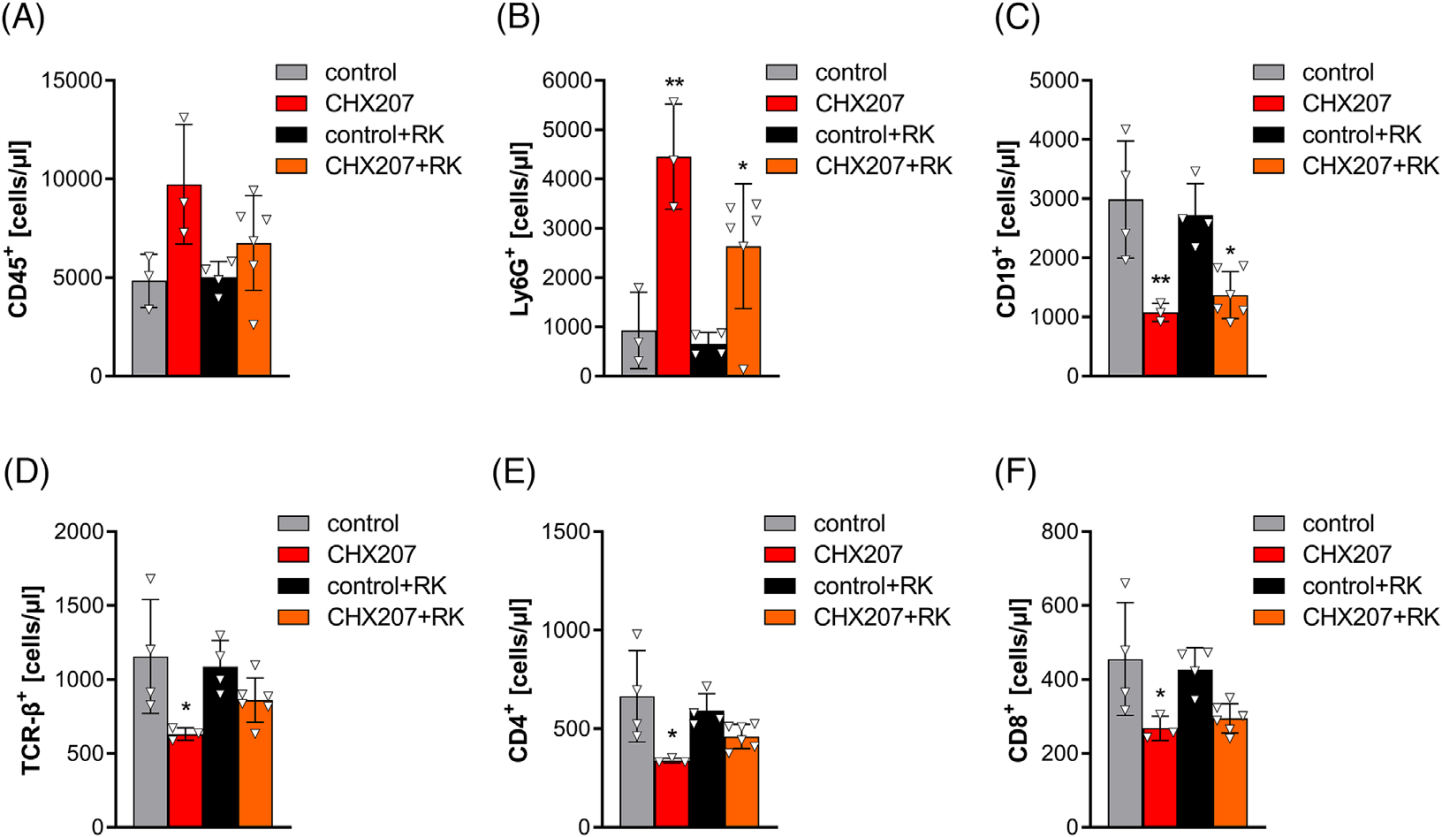
R‐ketorolac (RK) prevents CHX207‐induced T‐lymphopenia. Male C57BL/6J mice were inoculated with 1 × 10^6^ CHX207 cells (in 100 μL of phosphate‐buffered saline [PBS]) or PBS as control. (A–F) Blood was taken retro‐orbitally, and whole blood (anticoagulated with acid citrate dextrose) was stained using the indicated antibodies and analysed using a Cytek® Aurora spectral flow cytometer (*n* = 3 for CHX207, *n* = 3–4 for control and control + RK and *n* = 6 for CHX207 + RK mice). One‐way analysis of variance followed by Šidák's post hoc analysis was performed to identify statistical differences between the groups (*^/#^ *P* ≤ 0.05; **^/##^ *P* ≤ 0.01; ***^/###^ *P* ≤ 0.001, *control vs. C26 and control + RK vs. C26 + RK; ^#^control vs. control + RK and C26 vs. C26 + RK).

IL‐6 plasma concentrations were 93 ± 39 pg/mL in CHX207 mice and reduced to 35 ± 6 pg/mL by RK treatment (*P* = 0.0053). IL‐6 was not detected in the control groups (*Figure* *6* *F*). Blood‐cell analyses revealed a slight increase in circulating CD45^+^ and a drastic increase in Ly6G^+^ cells in PBS‐ (4.8‐fold) and RK‐treated CHX207 mice (4‐fold) compared with the respective controls (*Figure* *7* *A,B*). In contrast, CD19^+^ cells were lower in both PBS‐ (−63 ± 5%) and RK‐treated (−61 ± 4.6%) CHX207 mice compared with the respective control mice (*Figure* *7* *C*). TCR‐β^+^, CD4^+^ and CD8^+^ cells were reduced by 45.3 ± 3.6%, 49.2 ± 1.9% and 41.1 ± 7.2%, respectively, in CHX207 mice compared with control mice. RK treatment ameliorated cancer‐induced T‐lymphopenia (TCR‐β^+^: −27.5 ± 12.8%; CD4^+^: −20.8 ± 13.8%; and CD8^+^: −31.4 ± 12.9%; *P* > 0.05) (*Figure* *7* *D–F*). Together, these data demonstrate that RK is effective in ameliorating CAC by preventing severe lymphopenia and reducing IL‐6 blood concentrations in two different tumour models.

## Discussion

Recent advances in our understanding of the mechanisms underlying CAC have led to the discovery of potential drug targets to increase quality of life and life expectancy of cachectic cancer patients. These include tumour‐borne factors like leukaemia inhibitory factor (LIF),^S4^ zink‐alpha glycoprotein (ZAG),^S5^ tumour necrosis factor alpha (TNFα)^S6^ or growth differentiation factor 15 (GDF‐15).^S7^ Beta‐blocker,^S8^ anabolic and orexigenic agents like enobosarm^S9^ or anamorelin^S10^ have been proven valuable to ameliorate weight loss in mouse models of CAC and in off‐label clinical use.^15^ Chronic inflammation is a main feature of CAC, rendering anti‐inflammatory agents such as NSAIDs as potential therapeutics. Pre‐clinical studies and clinical trials targeting inflammation with COX inhibitors, such as celecoxib, ibuprofen, indomethacin or naproxen (reviewed in [16]), have shown inconsistent outcomes, varying from no effect to beneficial effects on survival and body weight in cachectic patients and animal models. The lack of approved drugs to treat CAC is due to side effects that are likely not tolerated by patients undergoing cancer therapy and/or their poor beneficial effect on life quality overall.

Ketorolac is a commonly used NSAID prescribed after surgery.^5^ Like many NSAIDS, ketorolac is also marketed as racemate. Enantiomers of NSAIDs often exhibit stereoselective pharmacologic and toxicologic differences.^17^ Likewise, the S‐form of ketorolac has been shown to exert 100‐fold higher inhibitory activity towards COX enzymes and 33‐fold higher ulcerogenic potential, compared with the R‐form in rats.^6^ Ketorolac undergoes little or no chiral inversion in humans; however, in mice, there is a chiral inversion from S‐ to R‐ketorolac but not from R‐ to S‐ketorolac.^18^ Accordingly, studies using the racemic form of the drug in mice primarily demonstrate the impact of the R‐enantiomer.

Here, we show that the R‐enantiomer of ketorolac (RK) could be considered as a drug to treat weight loss in patients suffering CAC. RK treatment ameliorated body weight loss by reducing adipose tissue and skeletal muscle loss in C26 and CHX207 tumour‐bearing mice. Importantly, cachectic C26 mice tolerated a combinatory treatment of RK and chemotherapy, achieving a 100% survival rate. As a substantial number of CAC patients show cachexia symptoms before they are diagnosed with cancer,^2^ halting body weight loss in already cachectic animals, as achieved with RK, is desirable. However, in future studies, we will investigate whether RK treatment can also prevent CAC development in cancer patients.

We found that RK, at a dose of 2 mg/kg/day, stopped weight loss in tumour‐bearing mice without inhibiting COX activity. This is especially important as most NSAIDs are not recommended for long‐term use due to the side effects associated with COX‐1 inhibition. In accordance, no evidence for toxicity was reported in mice treated with 5 mg/kg/day RK for 25 days.^19^ Previous studies showed that the R‐enantiomer of ketorolac selectively inhibits Rac1 and Cdc42 and reduces tumour growth, invasion and metastasis.^11^, ^20^ We did not observe differences in tumour growth between vehicle and RK‐treated animals, and a selective Rac1 inhibitor did not ameliorate body weight loss in tumour‐bearing mice, indicating that RK ameliorates CAC independent of Rac1.

Anorexia is a common symptom in CAC. However, cancer‐induced body weight loss is only partially caused by reduced food intake and not reversible by increasing caloric uptake.^1^ Tumour‐bearing mice also ate slightly less compared with non‐tumour‐bearing controls. While RK treatment did not affect food intake of C26 mice, food intake of CHX207 mice was slightly increased by the drug. This tumour model‐specific difference might be due to different cancer entities (fibrosarcoma vs. colorectal cancer), due to the different genetic backgrounds of mice (C57BL/6J vs. Balb/c) and/or due to different anorexigenic signals transmitted to the brain.^21^ Additionally, C26 mice exhibited 4.8‐fold higher plasma IL‐6 concentrations than CHX207 mice in the absence and in the presence of RK, likely contributing to a more severe anorectic phenotype. IL‐6 can act on the brain to regulate appetite,^22^ and inhibition of IL‐6 signalling suppresses anorexia in tumour‐bearing mice.^23^ Alternatively, RK may increase food intake of tumour‐bearing mice by inhibiting brain anandamide hydrolysis.^24^ Further studies in different cancer models are needed to investigate the effect of RK on food intake.

RK‐mediated preservation of body weight was associated with an increase in circulating lymphocytes in C26 and CHX207 mice. An increased neutrophil‐to‐lymphocyte ratio caused by reduced lymphocyte (lymphopenia) and/or increased neutrophil (neutrophilia) counts is a commonly used biomarker for systemic inflammation and associates with weight loss in cancer patients^25^ and murine models of CAC.^12^, ^14^, ^26^ Lymphopenia is consistently reported as an adverse prognostic factor for progression‐free and overall survival in a variety of tumours.^27^ Although the actual mechanisms causing lymphopenia in cancer patients are unclear, they may include the presence of a general immunosuppressed condition caused by cancer, the anti‐cancer therapy, lympholytic cytokines that are released from the tumour or a combination of all. In line with previous studies,^28^ we also found that lymphocytes are not required for the onset of C26‐induced cachexia. While RK tended to reduce T‐cells in the blood of control mice, no further reduction of T‐cells was observed in RK‐treated tumour‐bearing mice, indicating that RK prevents cancer‐induced lymphopenia. However, RK was completely ineffective in ameliorating CAC in thymus‐deficient nude mice, demonstrating that the beneficial effect of RK on body weight and survival in this model strictly depends on the presence of lymphocytes. Correcting lymphopenia with IL‐7^29^ has already been suggested to improve survival of cancer patients.^27^ We found reduced circulating CD4^+^ and CD8^+^ T‐cells in tumour‐bearing mice compared with controls and RK more efficiently increased CD4^+^ T‐cells than CD8^+^ T‐cells. Similarly, the severity of muscle atrophy caused by LL2 tumours^26^ and weight loss in mice infected with *Toxoplasma gondii* ^S11^ was significantly reduced in mice that received a CD4^+^ T‐cell infusion, demonstrating that protection from CD4^+^ T‐lymphopenia is associated with protection from cachexia.^26^ In contrast to its beneficial impact on lymphocyte numbers, RK treatment did not correct neutrophilia in C26 and CHX207 mice. Accordingly, recent studies indicate that neutrophilia is an adaptive response of the host against the cachexigenic tumour, and eliminating neutrophils aggravates weight loss in C26 mice.^14^

To date, we can only speculate on how RK affects circulating lymphocytes. Generally, NSAIDs are known to suppress T‐cell proliferation and activity.^S12^ ^,^ ^S13^ ^,^ ^30^ This immunosuppressant activity of NSAIDs on lymphocytes, however, depends on prostaglandin synthesis by COX‐1/2.^30^ As RK does not inhibit COX enzymes, its effect on lymphocyte number is likely different from NSAIDs. IL‐6 contributes to lymphopenia by initiating T‐cell pyroptosis^31^ and blocking IL‐6 reduced STAT3 activation in CD4^+^ T cells, demonstrating an IL‐6‐mediated regulation of lymphocytes.^32^ Accordingly, the beneficial effect of RK on cancer‐induced lymphopenia may partially be mediated by reducing IL‐6 concentrations.

While only little is known about the role of IL‐6 in cachexia‐associated lymphopenia, several studies demonstrated that IL‐6 signalling initiates weight loss in murine models of CAC.^12^, ^33^, ^34^, ^35^ IL‐6 plasma concentrations also correlate with poor treatment response and increased mortality in cancer patients.^36^ IL‐6, by binding to the soluble or membrane‐standing IL‐6 receptor, activates a STAT3‐dependent signal transduction cascade leading to the transcription of catabolic genes responsible for fat and muscle loss in mouse models of CAC.^12^, ^13^, ^33^, ^35^ RK more than halved circulating IL‐6 concentrations in tumour‐bearing mice and diminished STAT3 phosphorylation in adipose and muscle tissue, indicating that reducing systemic IL‐6 levels, and thereby limiting tissue STAT3 signalling, likely contributes to the beneficial effect of RK in tumour‐bearing mice. In accordance, protein and mRNA expression of genes associated with muscle atrophy was increased in muscles of C26 mice but reduced to control levels in C26 mice that received RK treatment. Counterintuitively, muscle fibre size was not increased by treating C26 mice with RK. Hence, the mechanisms underlying RK's effect on increasing muscle tissue weight in tumour‐bearing mice require further investigation. Previous studies demonstrated that the NSAIDs celecoxib and ibuprofen^S14^ as well as aspirin^S15^ reduce STAT3 phosphorylation in cancer cells. However, in vivo studies on systemic effects are still missing. Although drugs preventing IL‐6 signalling, such as suramin, tocilizumab or MR16‐1, inhibited C26‐,^37^ pancreatic cancer‐^35^ or LLC‐induced^38^ cachexia in mice, studies in humans were less convincing,^38^, ^39^ suggesting that in humans, a multifactorial systemic disease like CAC cannot be treated solely by targeting IL‐6 but by angling multiple mechanisms.

A limitation of our study is the use of syngeneic allografts instead of genetically engineered mouse models, which better reflect the naturally occurring pathophysiology of cancer. Moreover, in contrast to patients suffering from CAC, mouse models are only mildly anorectic. Therefore, the beneficial effect of RK on anorexia in CHX207 mice has to be considered with caution. Additionally, in‐depth toxicological studies are needed to ensure the drug's safety for long‐term use. Future studies in different models of CAC shall entangle how RK prevents cancer‐induced lymphopenia and reduces circulating IL‐6 concentrations and whether RK exerts its beneficial effects by modulating the abundance of other cachexokines.

Taken together, this study shows that RK ameliorates cachexia and prolongs survival of tumour‐bearing mice even upon chemotherapy. We propose that RK should be further investigated as a drug to treat CAC in humans.

## Funding

This research was funded in part by Yinuoke Ltd, the University of Graz and the Austrian Science Fund (FWF) [grant DOI: 10.55776/F83 and grant DOI: 10.55776/I5618]. For the purpose of open access, the authors have applied a CC BY public copyright licence to any Author Accepted Manuscript version arising from this submission.

## Conflict of interest statement

The authors of this manuscript declare that they have no conflict of interest.

## Supporting information


**Figure S1.** RK does not affect body weight of non‐tumor‐bearing and non‐cachexigenic tumor‐bearing mice. Balb/c mice were inoculated with **(B)** 1 × 10^6^ 4T1 cells (in 100 μl PBS) or **(A)** PBS (non‐tumor‐bearing mice). 2 mg/kg RK or PBS as control were administered daily by oral gavage. Body weight was determined over a period of 10 days (*n* = 4). Two‐way Anova followed by Šidák's *post hoc* analysis was performed to identify statistical differences between the groups.
**Figure S2.** RK does not inhibit Cyclooxygenase (COX) activity and Rac1 inhibition does not ameliorate C26‐induced cachexia. Male C57BL/6J mice were treated orally with 2 mg/kg/day RK for 5 consecutive days. One hour after the last dose, mice were sacrificed and COX activity was determined in lung homogenates using a Fluorometric COX Activity Assay (Abcam). (A) COX activity was determined by applying the slope to a standard calibration curve and is depicted per mg of lung protein. (B) Representative curves of Relative Fluorescence Units (RFU), measured during COX activity assay in the absence or presence of COX‐1/2 inhibitors (COXi; SC560/celecoxib) in lung homogenates of control and RK‐treated animals. (C, D) Male Balb/c mice were inoculated with 1 × 10^6^ C26 cells (in 100 μl PBS) or PBS as control. Mice were treated with (C) the Rac1 inhibitor NSC23766 (4 mg/kg), (D) RK (2 mg/kg) or PBS as control orally, every day, starting 2 days after cachexia onset (indicated with an arrow). Body weight was monitored for 4–6 additional days (*n* = 4). Two‐tailed unpaired t‐test (A) or two‐way Anova followed by Šidák's *post hoc* analysis (C, D) was performed to identify statistical differences between the groups. (*or# *p* ≤ 0.05; **or## *p* ≤ 0.01; ***or ### *p* ≤ 0.001, *control vs C26 and control + RK vs C26 + RK; #control vs control + RK and C26 vs C26 + RK).
**Figure S3.** Muscle tissue analyses. 12 days after C26 cancer cell inoculation and treatment with 2 mg/kg RK or PBS as control, blood was taken retro‐orbitally, mice were sacrificed by cervical dislocation, tissues were weighed and snap frozen in liquid nitrogen. (A, B) Muscle fiber areas were measured on H&E‐stained cross‐sections of m.quad. using CaseViewer (*n* = 3; >140 fibers per muscle). (A) Violin plot and (B) fiber size distribution. (C) Western Blotting analysis of skeletal muscle tissues using LC3B antibody and VINCULIN as loading control. Signal intensities were determined using Chemidoc (BioRad) and calculated using Image Lab (BioRad). (D, E) mRNA expression levels of marker genes for muscle catabolic signaling (D) *Fbxo32,* coding for Atrogin‐1, and (E) *Trim63,* coding for MuRF1, in skeletal muscles were determined by qRT‐PCR. *36b4* was used as housekeeping gene (*n* = 3–5). One‐way Anova followed by Šidák's *post hoc* analysis was performed to identify statistical differences between the groups (*or# *p* ≤ 0.05; **or## *p* ≤ 0.01; ***or ### *p* ≤ 0.001, *control vs C26 and control + RK vs C26 + RK; # control vs control + RK and C26 vs C26 + RK).

**Data S1.** Supplemental Methods.

**Data S2.** Supplemental References.
