# Supplementary material for: R‐ketorolac ameliorates cancer‐associated cachexia and prolongs survival of tumour‐bearing mice
Source: J Cachexia Sarcopenia Muscle. 2024 Feb 1;15(2):562–74. doi: 10.1002/jcsm.13422 (PMC10995265; doi:10.1002/jcsm.13422)
Supplement: Supplementary file 1 — Figure S1. RK does not affect body weight of non‐tumor‐bearing and non‐cachexigenic tumor‐bearing mice. Balb/c mice were inoculated with (B) 1 × 10^6^ 4T1 cells (in 100 μl PBS) or (A) PBS (non‐tumor‐bearing mice). 2 mg/kg RK or PBS as control were administered daily by oral gavage. Body weight was determined over a period of 10 days (n = 4). Two‐way Anova followed by Šidák's post hoc analysis was performed to identify statistical differences between the groups. Figure S2. RK does not inhibit Cyclooxygenase (COX) activity and Rac1 inhibition does not ameliorate C26‐induced cachexia. Male C57BL/6J mice were treated orally with 2 mg/kg/day RK for 5 consecutive days. One hour after the last dose, mice were sacrificed and COX activity was determined in lung homogenates using a Fluorometric COX Activity Assay (Abcam). (A) COX activity was determined by applying the slope to a standard calibration curve and is depicted per mg of lung protein. (B) Representative curves of Relative Fluorescence Units (RFU), measured during COX activity assay in the absence or presence of COX‐1/2 inhibitors (COXi; SC560/celecoxib) in lung homogenates of control and RK‐treated animals. (C, D) Male Balb/c mice were inoculated with 1 × 10^6^ C26 cells (in 100 μl PBS) or PBS as control. Mice were treated with (C) the Rac1 inhibitor NSC23766 (4 mg/kg), (D) RK (2 mg/kg) or PBS as control orally, every day, starting 2 days after cachexia onset (indicated with an arrow). Body weight was monitored for 4–6 additional days (n = 4). Two‐tailed unpaired t‐test (A) or two‐way Anova followed by Šidák's post hoc analysis (C, D) was performed to identify statistical differences between the groups. (*or# p ≤ 0.05; **or## p ≤ 0.01; ***or ### p ≤ 0.001, *control vs C26 and control + RK vs C26 + RK; #control vs control + RK and C26 vs C26 + RK). Figure S3. Muscle tissue analyses. 12 days after C26 cancer cell inoculation and treatment with 2 mg/kg RK or PBS as control, blood was taken retro‐orbitally, mice were sacri [file JCSM-15-562-s003.pdf]

Supplemental Figures and Legends

R-Ketorolac ameliorates cancer-associated cachexia and prolongs survival of tumor-bearing mice

Journal of Cachexia, Sarcopenia and Muscle

Sophia E. Chrysostomou[1], Sandra Eder[1], Isabella Pototschnig[1], Anna-Lena Mayer[1], Martina Derler[2], Marion Mussbacher[2], Silvia Schauer[3], Dongxu Zhang[5], Dongmei Yan[6], Gennie Liu[5], Gerald Hoefler[3], Thomas Weichhart[4], Paul W. Vesely[3], Lingbing Zhang[5]*, and Martina Schweiger[1,7,8]*

[1]Institute of Molecular Biosciences, University of Graz, Austria

[2] Institute of Pharmaceutical Sciences, University of Graz, Graz, Austra

[3]Diagnostic and Research Institute of Pathology, Medical University of Graz, Graz, Austria

[4]Institute of Medical Genetics, Medical University of Vienna, Vienna, Austria

[5]Yinuoke Ltd., Changchun, China

[6]Department of Immunology, Jilin University, Changchun, China

[7]Field of Excellence BioHealth - University of Graz, Graz, Austria

[8]BioTechMed-Graz, Graz, Austria

*Equal contribution of corresponding authors:

Martina Schweiger: tina.schweiger@uni-graz.at, Tel: +43 316 380 1908 and

Lingbing Zhang: lzhang@yinuokeus.com, Tel: 8613262666048

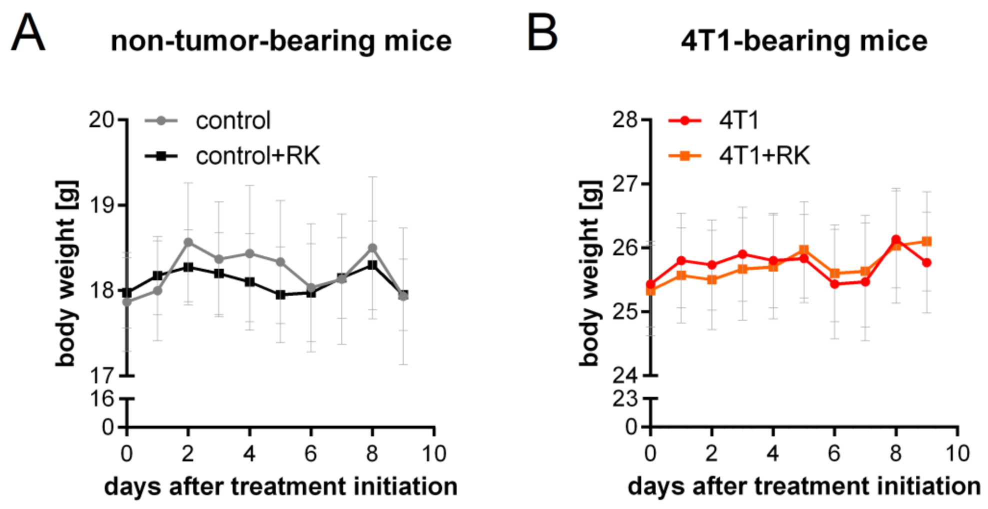

Supplemental Figure 1. RK does not affect body weight of non-tumor-bearing and non-cachexigenic tumor-bearing mice. Balb/c mice were inoculated with **(B)** $1x10^6$ 4T1 cells (in 100 µl PBS) or **(A)** PBS (non-tumor-bearing mice). 2 mg/kg RK or PBS as control were administered daily by oral gavage. Body weight was determined over a period of 10 days (n=4). Two-way Anova followed by Šidák`s *post hoc* analysis was performed to identify statistical differences between the groups.

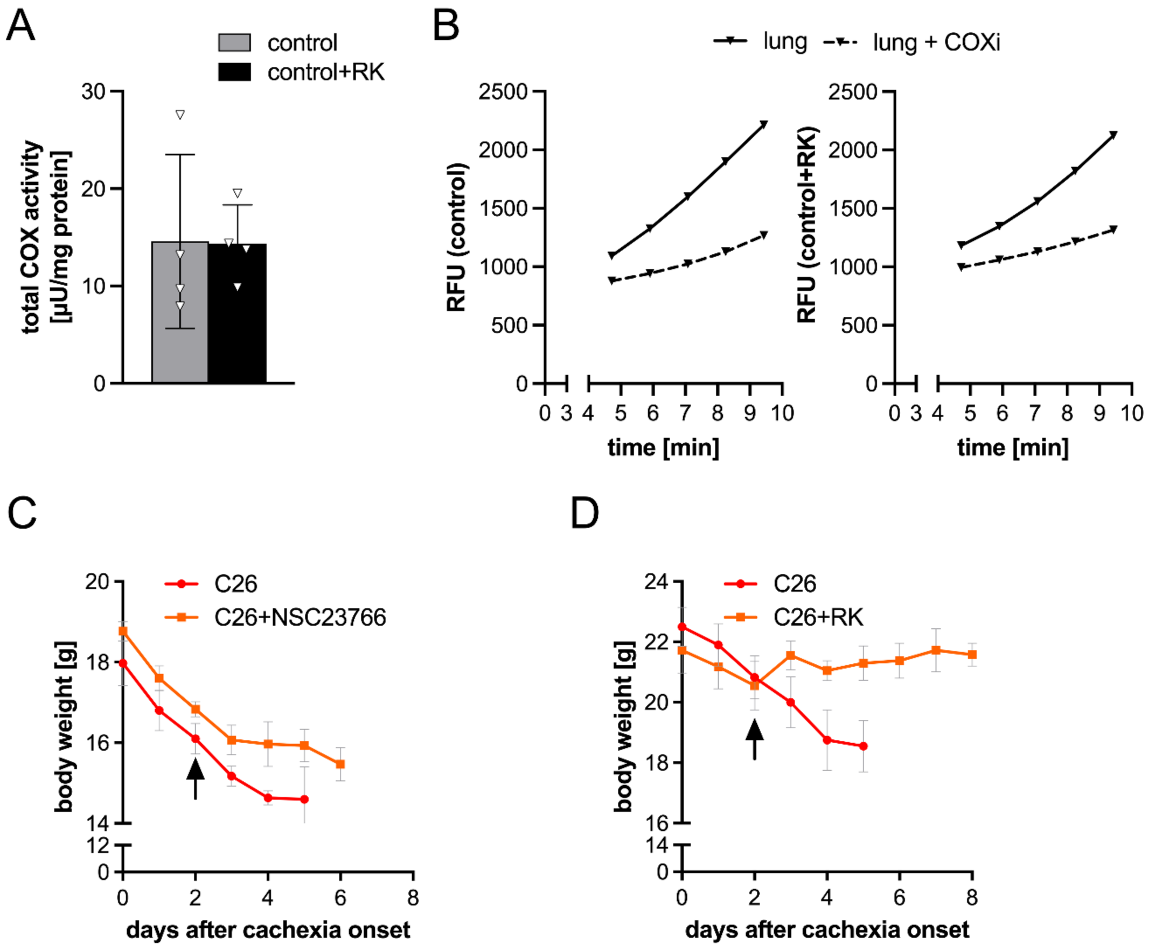

Supplemental Figure 2. RK does not inhibit Cyclooxygenase (COX) activity and Rac1 inhibition does not ameliorate C26-induced cachexia. Male C57BL/6J mice were treated orally with 2 mg/kg/day RK for 5 consecutive days. One hour after the last dose, mice were sacrificed and COX activity was determined in lung homogenates using a Fluorometric COX Activity Assay (Abcam). **(A)** COX activity was determined by applying the slope to a standard calibration curve and is depicted per mg of lung protein. **(B)** Representative curves of Relative Fluorescence Units (RFU), measured during COX activity assay in the absence or presence of COX-1/2 inhibitors (COXi; SC560/celecoxib) in lung homogenates of control and RK-treated animals. **(C, D)** Male Balb/c mice were inoculated with $1x10^6$ C26 cells (in 100 µl PBS) or PBS as control. Mice were treated with **(C)** the Rac1 inhibitor NSC23766 (4 mg/kg), **(D)** RK (2 mg/kg) or PBS as control orally, every day, starting 2 days after cachexia onset (indicated with an arrow). Body weight was monitored for 4-6 additional days (n=4). Two-tailed unpaired t-test (A) or two-way Anova followed by Šidák`s *post hoc* analysis (C, D) was performed to identify statistical differences between the groups. (*or# p≤0.05; **or## p≤0.01; ***or ### p≤0.001, *control vs C26 and control+RK vs C26+RK; #control vs control+RK and C26 vs C26+RK).

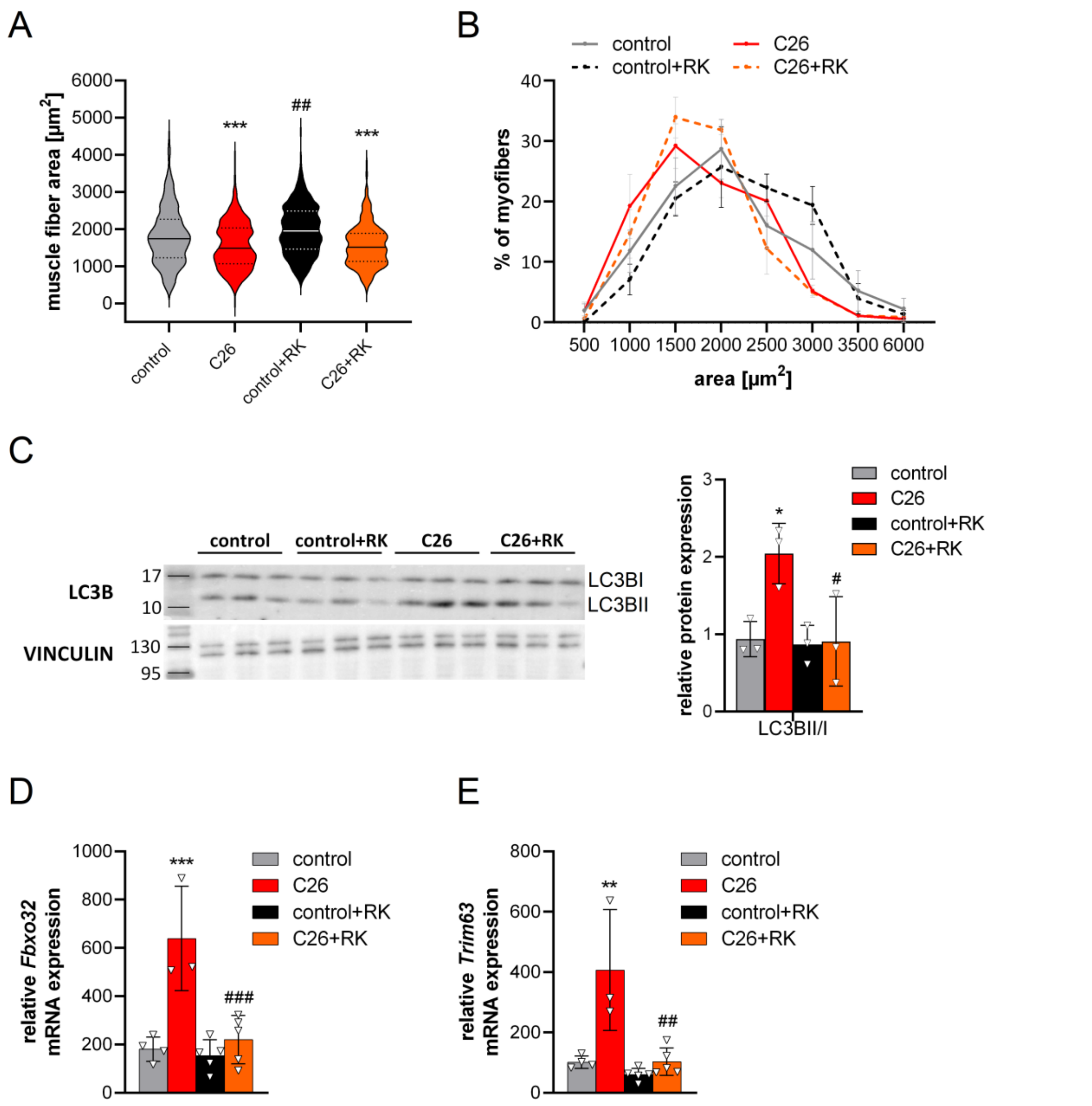

Supplemental Figure 3. Muscle tissue analyses. 12 days after C26 cancer cell inoculation and treatment with 2 mg/kg RK or PBS as control, blood was taken retro-orbitally, mice were sacrificed by cervical dislocation, tissues were weighed and snap frozen in liquid nitrogen. **(A, B)** Muscle fiber areas were measured on H&E-stained cross-sections of m.quad. using CaseViewer (n =3; >140 fibers per muscle). **(A)** Violin plot and **(B)** fiber size distribution. **(C)** Western Blotting analysis of skeletal muscle tissues using LC3B antibody and VINCULIN as loading control. Signal intensities were determined using Chemidoc (BioRad) and calculated using Image Lab (BioRad). **(D, E)** mRNA expression levels of marker genes for muscle catabolic signaling **(D)** *Fbxo32,* coding for Atrogin-1, and **(E)** *Trim63,* coding for MuRF1, in skeletal muscles were determined by qRT-PCR. *36b4* was used as housekeeping gene (n=3-5). One-way Anova followed by Šidák`s *post hoc* analysis was performed to identify statistical differences between the groups (*or$^{\#}$ p≤0.05; **or$^{\#\#}$ p≤0.01; ***or $^{\#\#\#}$ p≤0.001, *control vs C26 and control+RK vs C26+RK; $^{\#}$ control vs control+RK and C26 vs C26+RK).
