# Supplementary material for: R‐ketorolac ameliorates cancer‐associated cachexia and prolongs survival of tumour‐bearing mice
Source: J Cachexia Sarcopenia Muscle. 2024 Feb 1;15(2):562–74. doi: 10.1002/jcsm.13422 (PMC10995265; doi:10.1002/jcsm.13422)
Supplement: Supplementary file 2 — Data S1. Supplemental Methods. [file JCSM-15-562-s002.docx]

**Supplemental Methods**

**R-Ketorolac ameliorates cancer-associated cachexia and prolongs survival of tumor-bearing mice**

*Journal of Cachexia, Sarcopenia and Muscle*

Sophia E. Chrysostomou^1^, Sandra Eder^1^, Isabella Pototschnig^1^, Anna-Lena Mayer^1^, Martina Derler^2^, Marion Mussbacher^2^, Silvia Schauer^3^, Dongxu Zhang^5^, Dongmei Yan^6^, Gennie Liu^5^, Gerald Hoefler^3^, Thomas Weichhart^4^, Paul W. Vesely^3^, Lingbing Zhang^5^*, and Martina Schweiger^1,7,8^*

^1^Institute of Molecular Biosciences, University of Graz, Austria

^2^ Institute of Pharmaceutical Sciences, University of Graz, Graz, Austra

^3^Diagnostic and Research Institute of Pathology, Medical University of Graz, Graz, Austria

^4^Institute of Medical Genetics, Medical University of Vienna, Vienna, Austria

^5^Yinuoke Ltd., Changchun, China

^6^Department of Immunology, Jilin University, Changchun, China

^7^Field of Excellence BioHealth - University of Graz, Graz, Austria

^8^BioTechMed-Graz, Graz, Austria

Martina Schweiger: [tina.schweiger@uni-graz.at](mailto:tina.schweiger@uni-graz.at), Tel: +43 316 380 1908 and

Lingbing Zhang: [lzhang@yinuokeus.com](mailto:lzhang@yinuokeus.com)

**Antibodies used for flow cytometry:**

CD45 (30-F11; 1:50), CD11b (M1/70; 1:50), Ly6G (1A6; 1:200), CD19 (6D5; 1:200), TCR-β (H57-595; 1:50), CD4 (GK1.5; 1:200), and CD8 (53-6.7; 1:600); (Thermo Fisher Scientific).

**COX-activity assay:**

For COX-activity measurement 100 mg lung tissue were disrupted in 500 µl lysis buffer (1xPBS, 1% NP-40, 20 µg/ml leupeptin, 2 µg/ml antipain, 1 µg/ml pepstatin; Carl Roth GmbH, Karlsruhe, Germany) on ice using an Ultra-Turrax Homogenizer (IKA, Staufen, Germany) and centrifuged at 12,000 x g, 4 °C for 3 min. 5 µl lung cell lysate were mixed with a fluorometric probe and arachidonic acid. To inhibit COX1 and COX2 activity, SC560 and celecoxib, respectively, were added to the lysates according to the manufacturer’s protocol. COX-activity caused the production of a fluorescent molecule that was monitored in kinetic mode for 20 min at 37°C using a Glomax® Multimode Microplate Reader (Promega). Data were plotted as Relative Fluorescence Units (RFU) over time and COX activity was determined by applying the slope of the curve in its linear region to a resorufin standard calibration curve. Protein concentration of lung lysates was determined by Protein Assay Dye (Bio-Rad Laboratories, Hercules, USA).

**Western Blotting analysis.**

After SDS-PAGE, proteins were blotted onto a methanol-activated polyvinylidenfluorid (PVDF) membrane (Carl Roth GmbH) for 1 h at 200 mA. Unspecific binding sites were blocked using 5% milk powder or 5% BSA (Carl Roth GmbH) in 1xTST (10mM Tris, 150mM NaCl, 0.1% Tween 20) followed by incubation with a primary and an HRP-conjugated secondary antibody. HRP-conjugated antibodies were detected by chemiluminescence using Clarity Western Enhanced chemiluminescence (ECL) substrate (Bio-Rad Laboratories) and ChemiDoc Touch Imaging System (Bio-Rad Laboratories). Signal intensities were determined by densitometric analyses using Image Lab software (Bio-Rad Laboratories). Specific proteins were detected using following antibodies: VINCULIN (#V9131, 1:20000, Sigma Aldrich), pSTAT3 (#9145S, 1:1000, Cell signaling), STAT3 (#4904S, 1:1000, Cell signaling), and LC3B (#2775, Cell signaling). HRP-linked Anti-rabbit IgG antibody (#A120-201P, 1:10000, Bethyl, USA), HRP-linked Anti-mouse IgG antibody (#NA 931V, 1:10000, GE Healthcare, USA), and HRP-linked Anti-goat IgG antibody (#AP180P, Millipore, USA) were used as secondary antibodies.

**Primer sequences for qRT-PCR analysis:**

Atrogin-1fwd 5’CTTTCAACAGACTGGACTTCTCGA3’,

Atrogin-1rev5’CAGCTCCAACAGCCTTACTACGT3’;

MuRF1fwd 5’AGTGTCCATGTCTGGAGGTCGTTT3’,

MuRF1rev 5’ACTGGAGCACTCCTGCTTGTAGAT3’
