# Supplementary material for: R‐ketorolac ameliorates cancer‐associated cachexia and prolongs survival of tumour‐bearing mice
Source: J Cachexia Sarcopenia Muscle. 2024 Feb 1;15(2):562–74. doi: 10.1002/jcsm.13422 (PMC10995265; doi:10.1002/jcsm.13422)
Supplement: Supplementary file 3 — Data S2. Supplemental References. [file JCSM-15-562-s001.docx]

**Supplemental References**

**R-Ketorolac ameliorates cancer-associated cachexia and prolongs survival of tumor-bearing mice**

*Journal of Cachexia, Sarcopenia and Muscle*

Sophia E. Chrysostomou^1^, Sandra Eder^1^, Isabella Pototschnig^1^, Anna-Lena Mayer^1^, Martina Derler^2^, Marion Mussbacher^2^, Silvia Schauer^3^, Dongxu Zhang^5^, Dongmei Yan^6^, Gennie Liu^5^, Gerald Hoefler^3^, Thomas Weichhart^4^, Paul W. Vesely^3^, Lingbing Zhang^5^*, and Martina Schweiger^1,7,8^*

^1^Institute of Molecular Biosciences, University of Graz, Austria

^2^ Institute of Pharmaceutical Sciences, University of Graz, Graz, Austra

^3^Diagnostic and Research Institute of Pathology, Medical University of Graz, Graz, Austria

^4^Institute of Medical Genetics, Medical University of Vienna, Vienna, Austria

^5^Yinuoke Ltd., Changchun, China

^6^Department of Immunology, Jilin University, Changchun, China

^7^Field of Excellence BioHealth - University of Graz, Graz, Austria

^8^BioTechMed-Graz, Graz, Austria

*Equal contribution of corresponding authors:

Martina Schweiger: [tina.schweiger@uni-graz.at](mailto:tina.schweiger@uni-graz.at), Tel: +43 316 380 1908 and

Lingbing Zhang: [lzhang@yinuokeus.com](mailto:lzhang@yinuokeus.com), Tel: 8613262666048

S1. Bonetto A, Rupert JE, Barreto R, Zimmers TA. The Colon-26 Carcinoma Tumor-bearing Mouse as a Model for the Study of Cancer Cachexia. J Vis Exp 2016 doi:10.3791/54893.

S2. Tamilarasan KP, Temmel H, Das SK, Al Zoughbi W, Schauer S, Vesely PW et al. Skeletal muscle damage and impaired regeneration due to LPL-mediated lipotoxicity. Cell Death Dis 2012;3:e354–e354.

S3. Chapman J, Goyal A, Azevedo AM. Splenomegaly. StatPearls Publishing; 2022http://www.ncbi.nlm.nih.gov/pubmed/30638965. Accessed 20 February 2023.

S4. Kandarian SC, Nosacka RL, Delitto AE, Judge AR, Judge SM, Ganey JD et al. Tumour-derived leukaemia inhibitory factor is a major driver of cancer cachexia and morbidity in C26 tumour-bearing mice. J Cachexia Sarcopenia Muscle 2018;9:1109–1120.

S5. Elattar S, Dimri M, Satyanarayana A. The tumor secretory factor ZAG promotes white adipose tissue browning and energy wasting. FASEB J 2018;32:4727–4743.

S6. Chizzonite R, Lonnroth C, Lundholm K. Role of Endogenous Tumor Necrosis Factor a and Interleukin 1 for Experimental Tumor Growth and the Development of Cancer Cachexia. Cancer Res 1991;51:415–421.

S7. Suriben R, Chen M, Higbee J, Oeffinger J, Ventura R, Li B et al. Antibody-mediated inhibition of GDF15–GFRAL activity reverses cancer cachexia in mice. Nat Med 2020;26:1264–1270.

S8. Yuan L, Springer J, Palus S, Busquets S, Jové Q, Alves E et al. The atypical β -blocker S-oxprenolol reduces cachexia and improves survival in a rat cancer cachexia model. 2022;1–8.

S9. Dobs AS, Boccia R V, Croot CC, Gabrail NY, Dalton JT, Hancock ML et al. Effects of enobosarm on muscle wasting and physical function in patients with cancer: a double-blind, randomised controlled phase 2 trial. Lancet Oncol 2013;14:335–45.

S10. Temel JS, Abernethy AP, Currow DC, Friend J, Duus EM, Yan Y et al. Anamorelin in patients with non-small-cell lung cancer and cachexia (ROMANA 1 and ROMANA 2): results from two randomised, double-blind, phase 3 trials. Lancet Oncol 2016;17:519–531.

S11. Olguín JE, Fernández J, Salinas N, Juárez I, Rodriguez-Sosa M, Campuzano J et al. Adoptive transfer of CD4(+)Foxp3(+) regulatory T cells to C57BL/6J mice during acute infection with Toxoplasma gondii down modulates the exacerbated Th1 immune response. Microbes Infect 2015;17:586–95.

S12. Kazmi SM, Plante RK, Visconti V, Taylor GR, Zhou L, Lau CY. Suppression of NF kappa B activation and NF kappa B-dependent gene expression by tepoxalin, a dual inhibitor of cyclooxygenase and 5-lipoxygenase. J Cell Biochem 1995;57:299–310.

S13. Mortensen R, Clemmensen HS, Woodworth JS, Therkelsen ML, Mustafa T, Tonby K et al. Cyclooxygenase inhibitors impair CD4 T cell immunity and exacerbate Mycobacterium tuberculosis infection in aerosol-challenged mice. Commun Biol 2019;2:288.

S14. Moon H-J, Park S-Y, Lee S-H, Kang C-D, Kim S-H. Nonsteroidal Anti-inflammatory Drugs Sensitize CD44-Overexpressing Cancer Cells to Hsp90 Inhibitor Through Autophagy Activation. Oncol Res 2019;27:835–847.

S15. Zhang X, Feng H, Du J, Sun J, Li D, Hasegawa T et al. Aspirin promotes apoptosis and inhibits proliferation by blocking G0/G1 into S phase in rheumatoid arthritis fibroblast-like synoviocytes via downregulation of JAK/STAT3 and NF-κB signaling pathway. Int J Mol Med 2018;42:3135–3148.
